# How a Person Threatened or Afflicted with Bright’s Disease Ought to Live

**Published:** 1881-02

**Authors:** 


How a Person Threatened or Afflicted with Bright's Disease Ought to Live. By Joseph F. Edwards. Presly Blakiston; Philadelphia. 1881.

We have just received, through Messrs. J. J. & S. P. Richards, this little volume, whose object is to teach hygiene to that class of unfortunates who would be more benefitted, probably, could they be taught to accept with a proper resignation the finale which is, in by far the majority of instances, inevitable.

